# Multidrug-Resistant Tuberculosis—Diagnostic Procedures and Treatment of Two Beijing-like TB Cases

**DOI:** 10.3390/diagnostics12071699

**Published:** 2022-07-12

**Authors:** Monika Kozińska, Marcin Skowroński, Paweł Gruszczyński, Ewa Augustynowicz-Kopeć

**Affiliations:** 1 Department of Microbiology, National Tuberculosis and Lung Diseases Research Institute, Plocka 26, 01-138 Warsaw, Poland; e.kopec@igichp.edu.pl; 2 Wielkopolska Pulmonology and Thoracic Surgery Centre of Eugenia and Janusz Zeyland, Szamarzewskiego 62, 60-569 Poznań, Poland; mskowronski@wcpit.org (M.S.); pawel_gru@op.pl (P.G.)

**Keywords:** tuberculosis, drug resistance, *Mycobacterium tuberculosis*, Beijing-TB, Beijing-like genotype

## Abstract

The Beijing/W genotype is one of the major molecular families of *Mycobacterium tuberculosis* complex (MTBC), responsible for approximately 50% of tuberculosis (TB) cases in Far East Asia and at least 25% of TB cases globally. Studies have revealed that the Beijing genotype family is associated with a more severe clinical course of TB, increased ability to spread compared to other genotypes, and an unpredictable response to treatment. Based on the profile of spacers 35–43 in the Direct Repeat (DR) locus of the MTBC genome determined by spoligotyping, classical (typical) and modern (Beijing-like) clones can be identified within the Beijing family. While the modern and ancient Beijing strains appear to be closely related at the genetic level, there are marked differences in their drug resistance, as well as their ability to spread and cause disease. This paper presents two cases of drug-resistant tuberculosis caused by rare mycobacteria from the Beijing family: the Beijing 265 and Beijing 541 subtypes. The genotypes of isolated strains were linked with the clinical course of TB, and an attempt was made to initially assess whether the Beijing subtype can determine treatment outcomes in patients.

## 1. Introduction

Tuberculosis (TB) continues to be the communicable disease with the highest mortality rates in the world. Despite the implementation of Directly Observed Therapy (DOT), the global incidence of TB reduces by only 1–2% each year, which is well below the incidence estimated by mathematical models [1,2]. The main causes of this slow decline include, first of all, coinfection with HIV (Human Immunodeficiency Virus), malnutrition, drug resistance, low socioeconomic status, and inappropriate epidemiological surveillance of disease transmission [3,4]. The COVID-19 pandemic also significantly influenced the functioning of the public healthcare sector, serious epidemiological problems have been neglected, and the diagnostics targeted at many infectious diseases, including tuberculosis, have become less important [5]. Recently, clinicians, epidemiologists and microbiologists have had their attention drawn to the epidemiological risk posed by Beijing type tuberculosis (W/Beijing-TB) [6,7].

The Beijing genotype is characteristic for one of the seven major molecular families of *Mycobacterium tuberculosis* (Lineage 2, or East Asian Lineage), accounting for approximately 50% of *Mycobacterium tuberculosis* (MTB) strains isolated in Far East Asia and at least 25% of all MTB strains globally [8,9]. This genotype was first described in 1995 by Van Soolingen et al. as the dominant genotype of *Mycobacterium tuberculosis* circulating in the Chinese population. All strains isolated in 1992–1994 from this population of patients were characterized by the same pattern, RFLP-IS6110, with 15–20 copies of the insertion sequence and an atypical spoligotype compared to strains isolated in other regions of the world [10]. Isolates with such a DNA profile, cultured from patients in subsequent years, were defined as typical or classical Beijing, while clinical isolates containing only spacers 35–43 in the DR locus were named as atypical Beijing or Beijing-like [11]. Additional subdivision of the Beijing lineage is currently based on the presence of IS6110 insertions in the NTF chromosomal region and on the detection of alterations in putative mutator genes, mutT2 and mutT4. As described in the article by Ribeiro et al., the ancient (atypical) sublineage is characterized by an intact NTF region and the absence of changes in putative mutator genes [12].

The W/Beijing genotype is still considered endemic to China and its vicinity, but in other parts of the world, it has been the cause of TB epidemics more than once [13,14].

Studies have revealed that the Beijing family genotypes are associated with a more severe clinical course of TB, increased ability to spread compared to other genotypes, and an unpredictable response to treatment [15,16,17,18]. Importantly, some Beijing strains may be sensitive to antituberculotic drugs, but most of them are multidrug-resistant (MDR) [19,20,21]. Therefore, it is believed that patients infected with Lineage 2 mycobacteria should be carefully monitored in terms of diagnostic procedures and therapeutic outcomes [22] to eliminate the risk of developing drug resistance during therapy and to prevent the spread of these strains in the population.

The Beijing genotype has a highly conserved genome, which changes its nature less dynamically compared to other MTB genotypes. Moreover, many studies have described Beijing mycobacterial clones characterized by higher transmission rates and virulence compared to other subtypes [23,24].

In Poland, screening for Beijing type tuberculosis is carried out in the population of Poles, foreigners, and patients with drug-sensitive and drug-resistant tuberculosis. The first Polish report was published in 2010, and it described four cases of Beijing-TB caused by the multidrug-resistant Beijing 1 genotype isolated from a population of patients in 2004 [25]. A further study published in 2015 by Kozińska et al. reported that 71 patients with Beijing-TB were identified from 2007 to 2011 [21]. At that time, infection with both the ancient Beijing and the modern Beijing-like genotypes was identified in the study population.

This paper presents two cases of drug-resistant tuberculosis caused by rare mycobacteria from the Beijing family: the Beijing 265 and Beijing 541 subtypes. The genotypes of the isolated strains were linked with the clinical course of TB, and an attempt was made to initially assess whether the Beijing subtype can determine treatment outcomes in patients.

## 2. Case Study

### 2.1. 51-Year-Old Male Patient from Ukraine. MDR-TB, Beijing 541 Genotype, Successful Treatment

**Figure 1:**
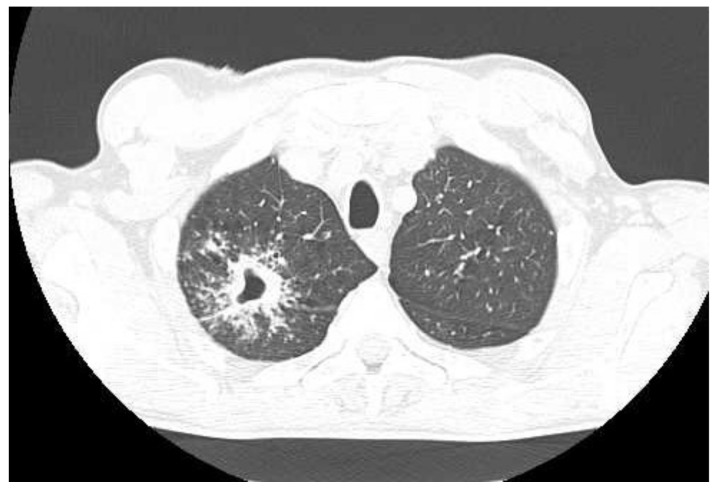
CT scan of the chest taken at the emergency department on admission to hospital in May 2021.

**Figure 2:**
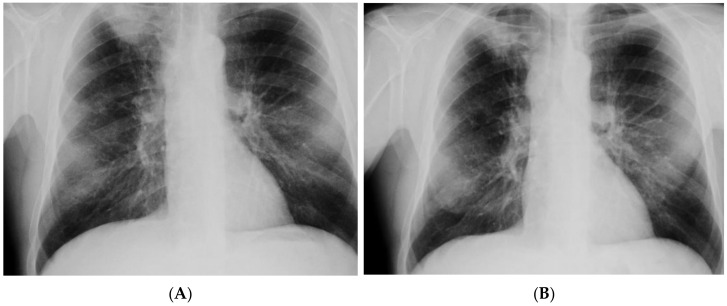
(**A**)—X-ray of the chest taken on 29 July 2021 (after 2 months of treatment)—irregular infiltration in the upper right field; (**B**)—12 October 2021 (after 5 months of treatment)—reduced infiltration.

**Figure 3:**
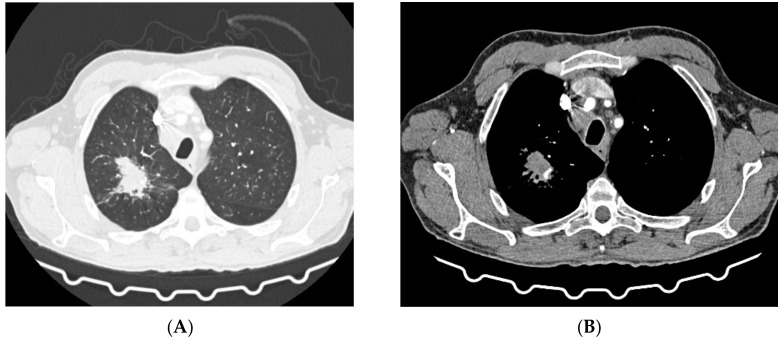
(**A**,**B**)—CT scans taken during the fifth month of treatment, September 2021.

In May 2021, the patient was admitted to the emergency department of a multispecialist hospital in Poznań for suspected pulmonary tuberculosis and multiple abscesses in the right supraclavicular region. One of them, the largest in size, had a large cutaneous fistula. A CT scan of the chest revealed two abscesses in the subcutaneous tissue in the right supraclavicular area, size 70 × 38 mm and 60 × 26 mm; a thick-walled cavity in the first right segment, size 24 × 28 mm with a contrasting capsule; a nodule diam. 13 mm, with a cavity inside, located in the right third segment; disseminated interstitial nodules up to 5 mm in diameter; and numerous enlarged mediastinal lymph nodes with contrasting capsules filled with a necrotic mass (Figure 1). Medical interview with the patient revealed a history of pulmonary tuberculosis, treated in 2015, and HIV infection. The patient had taken antiretroviral drugs in the past. He also reported tobacco and alcohol dependence. The patient was transferred to the tuberculosis department. His status was stable, with a productive cough, and was afebrile. Levels of inflammatory markers measured in laboratory tests were as follows: ESR 82 mm/h and CRP 8.1 mg/L. Due to the suspected tuberculosis, a sputum sample and abscess swab were collected for microbiological tests. The microscopic analysis of smears stained with the Ziehl–Neelsen (Z-N) technique revealed the presence of acid fast bacilli (AFB+++). MTBC DNA with a mutation in the *rpoB* gene was detected in both biological materials using the GeneXpert test (Cepheid, Synnyvale, CA, USA). According to the relevant protocol, the biological materials were cultured in Löwenstein–Jensen (LJ) solid medium and liquid medium (Becton Dickinson Diagnostic Systems, Sparks, MD, USA). The growth of MTBC was observed in cultures of sputum incubated at 37 °C for 5 days and in cultures of the abscess swab incubated for 8 days. The phenotypic method confirmed the resistance of isolates to antimycobacterial drugs: isoniazid (INH), rifampicin (RMP), ethambutol (EMB), streptomycin (SM), and rifabutin (RIF). In line with the recommendations for the treatment of multidrug-resistant tuberculosis (MDR-TB), defined as resistance to at least INH and RMP, the patient started therapy with second-line antituberculotic drugs in the following regimen: linezolid (LZD), cycloserine (CS), levofloxacin (LFX), ethionamide (ETO), clofazimine (CFZ), and pyrazinamide (PZA). The expanded antimicrobial susceptibility testing revealed that the isolated strains were sensitive to moxifloxacin (MFX), LZD, PZA, kanamycin (KM), amikacin (AM), capreomycin (CM), and CFZ. The strains were sent to the National Reference Laboratory for Tuberculosis (NRLT) in Warsaw for genotyping. Based on the spoligotyping technique (Ocimum Biosolutions, Hyderabad, India), both strains were classified to the Beijing type 541 family (spoligotype). The spoligotype and phylogenetic subtype were assigned according to the SITVIT WEB database (http://www.pasteur-guadeloupe.fr:8081/SITVIT_ONLINE/, accessed on 17 March 2022), administered by the Institute Pasteur de Guadeloupe (Table 1) [26]. Additional tests confirmed HIV infection by detecting anti-HIV antibodies and HIV-RNA (2.78 × 10^6^ IU/mL), but the test for the p24 antigen was negative. The count of CD4^+^ lymphocytes was 32 cells/mm^3^. After more than one month of antituberculotic therapy, three antiretroviral drugs were introduced: dolutegravir, emtricitabine, and tenofovir. The patient also received prophylactic treatment with azithromycin, sulfamethoxazole and trimethoprim. The treatment was well tolerated, and after one month the retest of a sputum smear collected from the patient was negative for *Mycobacterium*, and no MTBC were cultured. The chest X-ray performed after a two-month-long treatment showed an irregular infiltration in the upper right area of the lungs (Figure 2A). There was a gradual improvement in the general health of the patient, with weight gain of about 4 kg, reduction in coughing, and resolution of the abscess with a significant reduction in the fistula size. In the antimycobacterial treatment regimen, ETO was discontinued after 2 months. Another follow-up chest X-ray performed during the fifth month of treatment showed a marked reduction in the size of the oval infiltration at the apex of the right lung (Figure 2B). A CT scan of the chest revealed an oval nodular lesion with calcification, size 36 × 20 mm, in the right upper lobe, connected with the pleura via protrusions; enlarged cluster-like right mediastinal lymph nodes, with the involvement of the vessels and bronchi of the right hilum; enlarged left mediastinal nodes; nodular lesions (8 mm in the right upper lobe—anterior segment, and 7 mm in the left lower lobe of the apical segment); and multiple small nodules up to 2 mm in diameter (Figure 3A,B). Bronchofiberoscopy revealed a flat infiltration of the mucosa with superficial necrosis in the lower lobe bronchus. Biopsy specimens were collected for histopathological analysis which revealed squamous cell lung carcinoma (p40+, TTF1−, CK7−). In the follow-up test after 4 months, the count of CD4^+^ lymphocytes was 70 cells/mm^3^. Oncological treatment was postponed because the patient had no valid health insurance. During the 6-month hospitalization, the patient voluntarily left the tuberculosis department and discontinued the treatment. The patient was located and admitted to the internal medicine department at another hospital, where he completed antimycobacterial treatment, was discharged, and referred for further oncological treatment in Ukraine.

### 2.2. 58-Year-Old Male Patient. Pre-XDR-TB, Beijing 265 Genotype, Unsuccessful Treatment

**Figure 4:**
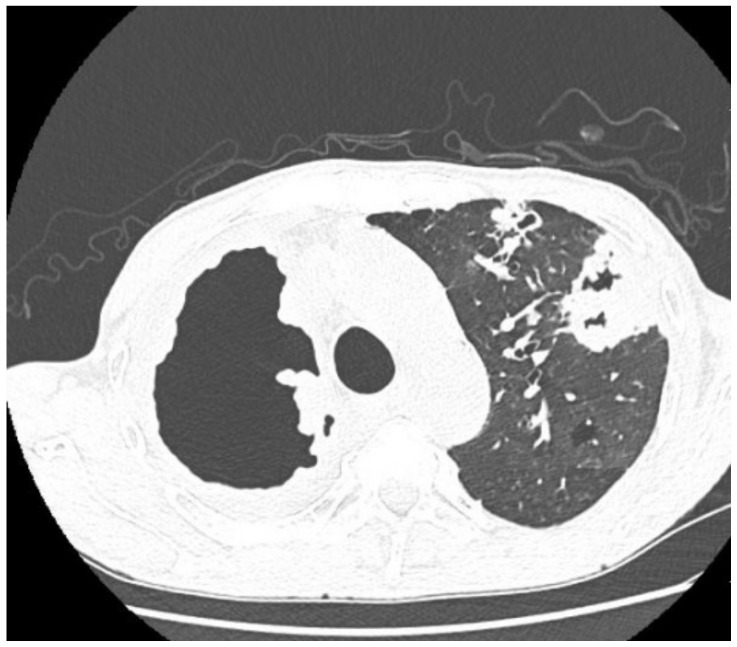
CT scan of the chest taken at the beginning of treatment 21 December 2020.

In April 2020, he was transferred to the Tuberculosis Department of the Wielkopolska Pulmonology and Thoracic Surgery Centre from another specialist pulmonary hospital for the treatment of pulmonary tuberculosis, confirmed by positive staining (Z-N) of a sputum smear (AFB+++) and MTBC culture. For the previous 2 months, the patient had been on the antimycobacterial treatment regimen: RMP, INH, PZA, EMB, and SM. When interviewed, the patient reported a productive cough, loss of appetite and approx. 16 kg weight loss. In 2019, he was treated for pulmonary tuberculosis sensitive to first-line antimycobacterial drugs. The patient reported that he took the prescribed medications irregularly, did not come to the follow-up visits at the outpatient clinic, and did not complete the treatment. Additionally, he reported tobacco and alcohol dependence. On admission to the hospital, the patient was emaciated, and his BMI (Body Mass Index) was 17 kg/m^2^. Laboratory tests revealed increased levels of inflammatory markers: ESR 90 mm/h, CRP 136, and anemia HGB 6.6 mmol/L). Microbiological analysis of a sputum smear was positive (AFB+++), and then the GeneXpert system confirmed the presence of genetic material from MTBC with a mutation in the *rpoB* gene. Four days after the incubation of the sputum culture the growth of MTBC strain resistant to INH, RMP, PZA, RIF, and SM was observed. Resistance of the strain to RMP, INH, and fluoroquinolones (FLQ) was detected using a molecular assay for antimicrobial susceptibility testing (Hain Lifescience GmbH, Nehren, Germany). The patient was treated with second-line antimycobacterial drugs according to WHO recommendations in the following regimen: CFZ, LZD, LFX, CS, EMB, and ETO. Antimicrobial susceptibility testing was repeated using a phenotypic method and liquid growth medium, the Bactec MGIT 960 system (Becton Dickinson Diagnostic Systems, Sparks, MD, USA). Resistance to ofloxacin (OFX), SM, INH, RMP, PZA, and RIF was detected, based on which the strain profile was defined as pre-extensively drug resistant (pre-XDR; MDR with concomitant resistance to any FLQ [27]. Based on a spoligotyping assay carried out at NRLT, the strain was identified as Beijing type 265 (Table 1). The therapy was continued, but due to a decrease in the patient’s appetite, it was interrupted periodically. A follow-up culture was established on 19 November 2021 and the growth of MTB Beijing 265 strain was observed again, but it was additionally resistant to KM, LZD, and EMB. Contrast-enhanced CT of the chest performed on 21 December 2021 revealed an extensive cavity in the upper lobe of the right lung, infiltrative lesions in the middle lobe, and smaller cavernous lesions in the left lung (Figure 4). 

**Figure 5:**
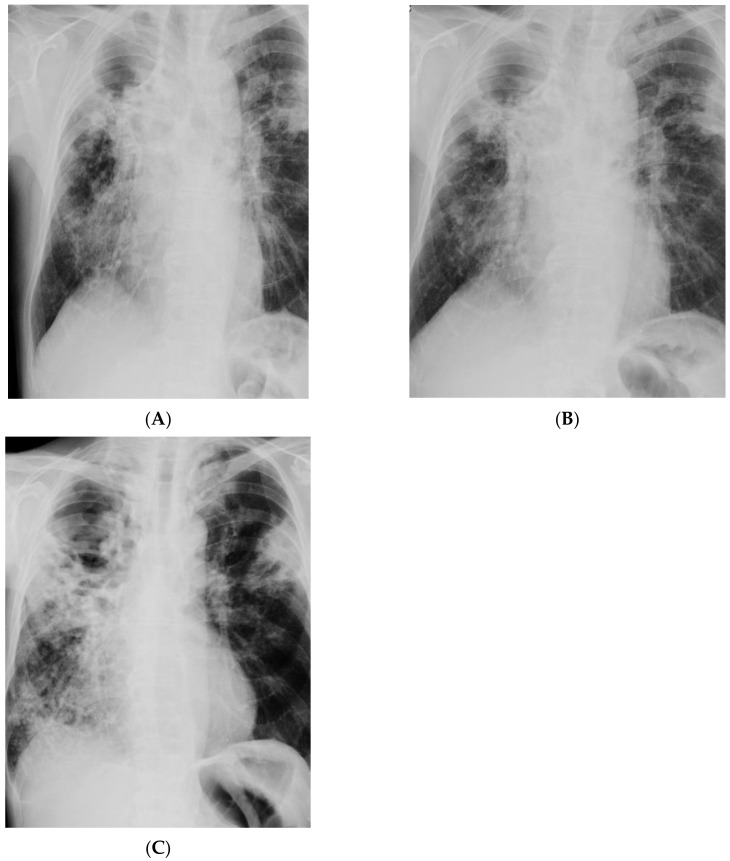
(**A**)—X-ray of the chest taken on admission to hospital. Chest X-ray showed extensive bilateral infiltrative lesions and a large thick-walled cavity in the upper right area (size 114 × 83 mm); (**B**)—X-ray taken after 17 months of treatment, no radiological improvement; (**C**)—X-ray taken in April 2022, no radiological improvement after 2 years of treatment. There are diffuse, blotchy, confluent dense areas in the parenchyma covering the upper and middle areas of the right lung and the middle area of the left lung. Some fibrous component of lesions distorting the shape of the hila. A cavity, 83 mm in diameter, present in the apical area of the right lung. A cavity, 24 mm in diameter, in the middle area of the left lung.

Despite more than 17-month-long antimycobacterial treatment, MTBC were still detected in sputum by microbiological analysis, the sputum smear was positive for mycobacteria (AFB+++), and culture growth was observed. Reduction in inflammatory markers was achieved: ESR 44 mm/h, CRP 35 mg/L. Compared to the X-ray examinations performed on admission to the hospital (Figure 5A), there was no improvement by radiographic criteria (Figure 5B). The patient’s health was stable during treatment, and he showed a good tolerance of antimycobacterial drugs. The patient’s body weight increased by 4 kg. Bedaquiline (TMC-207) was included in the treatment regimen. Treatment of TB was continued in a hospital setting, but to date, the patient is still positive for MTBC. The last radiogram of the chest taken in April 2022 is presented in Figure 5C.

**Table 1:** Microbiological and clinical characteristics of the reported cases.

	Patient 1	Patient 2
Sex	Male	Male
Age	51	58
Origin	Ukraine	Poland
Comorbidities	HIV Nicotine dependence Alcohol dependence	Nicotine dependence Alcohol dependence
Symptoms on admission to hospital	Stable; productive cough An abscess with a large cutaneous fistula in the right supraclavicular area	Productive cough, cachexia, lack of appetite, 16 kg weight loss
**TB**		
Medical history	2015—Pulmonary tuberculosis	2019—Pulmonary tuberculosis, drug-susceptible, treated irregularly, no follow-up visits, treatment discontinued
Imaging studies	Chest CT: Lesions in the lungs (cavities and nodules), enlarged mediastinal lymph nodes, 2 abscesses in the subcutaneous tissue in the right supraclavicular area	Chest X-ray: Extensive bilateral infiltrative lesions, large cavity in the upper area of the right lung (Figure 5A)
Bacteriological assay	Sputum AFB (+++)—MTBC cultured; Abscess swab—AFB (++)—MTBC cultured;	Sputum AFB (+++)—MTBC cultured
Genetic assay	GeneXpert Sputum (+), mutation in the *rpoB* gene; Abscess swab (+), mutation in the *rpoB* gene	GeneXpert Sputum (+), mutation in the *rpoB* gene
Drug resistance profile	MDR Phenotypic resistance to SM, INH, RMP, EMB, RIF	Pre-XDR Molecular resistance to INH, RMP, FLQ, KM; Phenotypic resistance to INH, RMP, OFX, PZA, RIF, SM, KM
Spoligotyping	Beijing 541 □□□□□□□□□□□□□□□□□□□□□□□□□□□□□□□□□□■■■■■□□■■	Beijing 265 □□□□□□□□□□□□□□□□□□□□□□□□□□□□□□□□□□■■□■■■■■■
TB treatment	PZA, LZD, CS, LFX, ETO, CFZ	EMB, LZD, CS, LFX, ETO, CFZ, AMK, TMC-207
**Imaging studies during antimycobacterial treatment**	Chest X-ray: After 2 mo of treatment—an irregular infiltration in the upper area of the right lung (Figure 2A). After 5 mo of treatment—reduced infiltration (Figure 2B) Chest CT: After 5 mo of treatment an oval nodular lesion with calcification, enlarged right and left mediastinal nodes, nodular lesions in the lungs (Figure 3A,B)	Chest X-ray: No improvement by radiographic criteria after 17 mo of treatment (Figure 5B). Still no improvement by radiographic criteria after further 2 years of treatment (Figure 5C) Chest CT: An extensive cavity in the upper lobe of the right lung, infiltrative lesions in the middle lobe, cavernous lesions in the left lung (Figure 4)
**Additional tests performed during hospitalization**	anti-HIV antibodies (+), HIV-RNA (+), p24 antigen (−), CD4^+^ lymphocytes—32 cells/mm^3^Bronchofiberoscopy: Histopathological analysis—squamous cell lung carcinoma (p40+, TTF−, CK7−)	No data
**Course and outcome of** **treatment**	Improved general health, 4 kg weight gain, reduction of cough, abscess resolution and reduction in fistula size; Recovery from TB, oncological treatment postponed	Therapy periodically interrupted; Still no recovery from TB after 2 years of treatment

## 3. Discussion

Until recently, it was generally believed that TB was caused by a pathogen with uniform characteristics, since its genome was considered to be highly conserved and without horizontal gene transfer [22]. However, studies have demonstrated that small mutations and changes at the regulatory level, often caused by selective environmental pressure and the interaction with the host organism, might induce significant changes in the mycobacterial phenotype. Experiments on animal and cell models and population studies have revealed the existence of hyper-, hypo- and mildly virulent MTBC strains and their different resistance to antibiotics [24,28,29,30,31]. Such differences in virulence and drug resistance profile of mycobacteria have a negative impact on the efficacy of treatment, making it difficult to achieve the goal of reducing TB incidence and mortality, especially from Beijing strains.

In Poland, patients with Beijing-TB are diagnosed each year [21,25,32]. By using spoligotyping for MTBC identification, it was possible to track changes in the molecular structure of mycobacteria isolated from patients in Poland. Spoligotyping enabled the identification of TB patients shedding lineage 2 mycobacterial strains of different phylogenetic subtypes. Until now, many Beijing-TB epidemics, caused by the ancient Beijing 1 genotype, have been reported around the world, and therefore, it was possible to better understand and characterize this pathogen. Its increased virulence, multidrug resistance, and the cause of treatment failure and TB relapse have been proven [15,18]. Today, the Beijing 1 genotype accounts for approx. 93% of all Beijing strains registered in the global database (source: http://www.pasteur-guadeloupe.fr:8081/SITVIT_ONLINE/, accessed on 17 March 2022).

Still, little is known about tuberculosis caused by rare Beijing (Beijing-like) genotypes [33]. Findings reported to date suggest that Beijing sublineages may evolve into highly pathogenic clones. However, there are no population studies or demographic, epidemiological or clinical data on patients with Beijing-like TB, and the only source providing selective information is SITVIT, an online *Mycobacterium tuberculosis* molecular markers database.

In most countries, modern Beijing strains are much more widespread than the ancient ones, which suggests their active spread. The exception is Japan, where the ancient W genotype is dominant [34]. Although the modern and ancient Beijing strains appear to be closely related at the genetic level, there are marked differences in their drug resistance profiles and their ability to spread and cause disease [23,35]. Moreover, the increased ability of Beijing strains to circumvent the immunity induced by the BCG vaccine has been proven, and studies by Kremer et al. have demonstrated that modern Beijing-like strains are isolated more often from BCG vaccinated patients than from unvaccinated individuals [17,18,36]. The observation that the interaction of modern Beijing strains with the host’s immune system is different from that of the ancient genotypes was further confirmed by van Laarhoven et al., who described differences in the induction of pro-inflammatory cytokines for both Beijing sublineages [37].

In this paper, we presented two cases of patients infected with Beijing-like strains representing subtypes 541 and 265. At the time of this analysis, in the SITVIT2 online spoligotype database, there were 16 MTB strains with the Beijing 541 genotype and 81 isolates with the Beijing 265 genotype, which accounted for 0.1% and 0.7%, respectively, of all Beijing strains registered worldwide (n = 10,850). The occasional isolation of atypical Beijing mycobacteria from patients means that little is known about the effect of these pathogens on the course of treatment and prognosis. Only a few relevant reports have been published, including two from Colombia, describing cases of tuberculosis caused by the modern Beijing subtype 190 mycobacteria [38,39]. Another report described the case of a 15-year-old female patient infected with this subtype of multidrug-resistant MT who died after unsuccessful treatment [40]. The first patient described in our report was infected with Beijing type 541 mycobacteria, and despite the multidrug resistance of the strain, therapeutic success was achieved. The second patient, infected with the Beijing 265 strain, is still AFB-positive despite long-term antimycobacterial therapy, which is confirmed by the results of microbiological tests carried out at the beginning of 2022 (MTBC culture, January 2022).

Our study has some limitations because although treatment failure is generally more common in patients with Beijing-type TB, there are a number of other factors that may additionally contribute to this, such as ethnicity, age, and sex [41,42]. It is not entirely possible to conclude whether treatment failure is associated with the genotype or with the drug resistance of mycobacteria, inappropriate adherence to the therapeutic regimen, or other factors that make the problem complex. Therefore, findings from our study must be interpreted with caution, as they do not clearly explain whether the high virulence and multidrug resistance of the Beijing 265 strain observed in our study is only episodic or is a feature closely related to this specific genotype.

## 4. Conclusions

*Mycobacterium tuberculosis* Beijing strains are particularly important in the surveillance of the spread of TB. The growing share of drug-resistant clones in this molecular family may significantly affect the global epidemiological situation and hinder the eradication of TB. It is therefore necessary to actively monitor the incidence of Beijing-TB and to implement effective methods of preventing its transmission. Considering the fact that the Beijing family includes subfamilies that are more virulent than others, the subtype of isolated Beijing strains should also be identified. Subtyping is also essential because studies have demonstrated a relationship between the Beijing subtype, drug resistance, disease course and treatment outcome.

## Data Availability

The results of the presented research are archived in the documentation of Wielkopolska Pulmonology and Thoracic Surgery Centre of Eugenia and Janusz Zeyland, Szamarzewskiego 62, 60-569 Poznań, Poland. Microbiological documentation has been archived at the Department of Microbiology, National Tuberculosis and Lung Diseases Research Institute, Plocka 26, 01-138 Warsaw, Poland.
